# Diagnosis and phenotypic assessment of Pakistani Haemophilia B carriers

**DOI:** 10.12669/pjms.333.12496

**Published:** 2017

**Authors:** Muhammad Tariq Masood Khan, Arshi Naz, Jawad Ahmed, Tahir Sultan Shamsi, Abid Sohail Taj

**Affiliations:** 1 Dr. Muhammad Tariq Masood Khan, MBBS, PhD Scholar. Institute of Basic Medical Sciences, Khyber Medical University, Peshawar, Pakistan; 2 Dr. Arshi Naz, PhD. National Institute of Blood Diseases & Bone Marrow Transplantation, Karachi, Pakistan; 3 Dr. Jawad Ahmed, PhD. Institute of Basic Medical Sciences, Khyber Medical University, Peshawar, Pakistan; 4 Dr. Tahir Sultan Shamsi, MRC Path, FRC (Path), National Institute of Blood Diseases & Bone Marrow Transplantation, Karachi, Pakistan; 5 Dr. Abid Sohail Taj, PhD, MRC (Path), National Institute of Blood Diseases & Bone Marrow Transplantation, Karachi, Pakistan

**Keywords:** Carriers, Factor IX, Haemophilia B, Linkage Analysis

## Abstract

**Objectives:**

1: To assess the diagnostic utility of three polymorphisms (DdeI, XmnI and TaqI) and direct sequencing in haemophilia B (HB) carrier detection in Pakistani families. 2: To compare phenotypes of HB carriers with those of healthy females.

**Methods:**

The study was conducted from March 2014 till February 2016 at Khyber Medical University Peshawar and National Institute of Blood Diseases, Karachi. Individuals from HB families of Khyber Pakhtunkhwa (KP) and Federally Administered Tribal Areas (FATA) with known F9 mutation in the proband were enrolled into the study. FIX activity (FIX: C) levels were determined in all the participants. Bleeding scores (BS) and complete blood counts were performed in the female participants. Linkage analysis followed by targeted Sanger sequencing was carried out in all the study participants. Heterozygosity rate was determined for each polymorphism. Healthy females and the carrier groups were compared for bleeding phenotypes.

**Results:**

A total of 30 males and 48 females from 13 HB families were studied. The polymorphisms had a low heterozygosity rate. Direct sequencing determined the carrier status in all cases. The mean FIX: C was reduced whereas BS was raised in the carriers when compared with healthy females. A significant raise in white blood cells (WBCs) count was observed in the carriers.

**Conclusion:**

The three polymorphisms have a low heterozygosity rate in HB families from KP and FATA. Sanger sequencing is conclusive in determining carrier status in all the cases. FIX: C is low and BS is raised in the HB carriers in comparison to that of normal females. The mean WBCs count is significantly higher in the HB carriers than the normal females.

## INTRODUCTION

Factor IX (FIX) is a coagulation factor with a pivotal role in the amplification and propagation phases of coagulation process.^1^ A deficiency in FIX activity (FIX:C) causes haemophilia B (HB). The FIX gene (*F9*) (MIM: 300746) has 8 exons and is approximately 32.7kbp long. A number of polymorphisms have been reported in this gene.^2^ Of particular interest are the restriction fragment length polymorphisms (RFLPs). These restriction sites include one exonic (*MnlI* in exon 6), three intronic (*Xmn*I in intron 3, *Taq*I and *Msp*I in intron 4), two in the 5’ flanking region (*MSe*I and *BamH*I) and one in the 3’ untranslated region (UTR) (*Hha*I). Two other unique complex repeats are found in intron 1 (*HinfI/DdeI)* and in 3’ UTR. These polymorphisms can be employed to determine the inheritance pattern of *F9* alleles within families that can thus help in carrier identification.^3^

Capillary electrophoresis is the current gold standard for mutation analysis and carrier identification in haemophilia.^4^ With the significant decline in cost in recent years, the facility is increasingly being adopted in developing countries as well.^5^ However, the classic mode of carrier testing, i.e. linkage analysis is still largely in practice. The test is considerably cheaper but it is cumbersome, time consuming and inconclusive at times.^6^ The two tests, together with pedigree analysis can be used cost-effectively for prenatal diagnosis and carrier identification in HB families.^7^

To date, only one study has been conducted on carrier identification in Pakistani HB families.^8^ The study employed linkage analysis, which was non-conclusive in about 50% of the cases. The five polymorphisms used included *DdeI* which is previously reported to be the most prevalent among neighboring Indian population.^9^ The current study reconfirms the heterozygosity rate of *DdeI* in Pakistani HB population of Khyber Pakhtunkhwa (KP) province and Federally Administered Tribal Areas (FATA). Heterozygosity status for two other polymorphisms, previously non-tested in Pakistani population, is also determined. The study further incorporates pedigree analysis and Sanger sequencing to determine the carrier status in HB families. It also portrays a phenotypic comparison between HB carriers and healthy females from the population, a subject unexplored in Pakistani population.

## METHODS

### Study duration and settings

The current study was conducted from March 2014 till February 2016 at Khyber Medical University Peshawar and National Institute of Blood Diseases, Karachi.

### Study participants

HB families from KP and FATA with F9 mutation previously identified in the index patient were selected. All consenting family members were enrolled into the study after acquisition of informed consent in writing. Those with chronic medical illnesses or with acute infections in the past two weeks were excluded. Female participants using hormonal contraceptives were also not included into the study.

### Preliminary data and bleeding scores

A family pedigree was constructed for each family and a comprehensive questionnaire depicting demographic and clinical details of the study participants was filled out. Bleeding scores were determined in all the female study participants using International Society on Thrombosis and Haemostasis Bleeding Assessment Tool (ISTH-BAT).^10^

### Sample collection and haematological investigations

Blood was collected from all female participants in a 2.7ml Plastic Citrate Tube (catalog # 363083; BD Vacutainer^®^) and in a 2ml K2EDTA containing plastic tube (catalog # 367841; BD Vacutainer^®^). Among women of child bearing age, it was collected only in the inter-menstrual period. A complete blood count (CBC) of the collected samples was performed on Sysmex pocH-100i^®^ Automated Hematology Analyzer (Sysmex Corporation, Kobe, Japan). The sample was then stored at 2-8°C until further analysis.

### Haemostasis Investigations

Platelet poor plasma was extracted from citrated blood. Prothrombin time (PT), activated partial thromboplastin time (APTT) and FIX: C assay were performed on STA COMPACT MAX^®^ (STAGO, US) haemostasis analyzer. Some of the results were cross checked on SYSMEX CA 1500^®^ coagulation analyzer (Kobe, Japan) to establish reliability.

### Linkage analysis

Three intragenic RFLPs were studied in all the study participants. Description of the selected polymorphisms along with the primers used is given in Table-I.

**Table-I:** RFLP primers for linkage analysis in HB families.

*Site*	*Restriction Enzyme*	*Forward Primer/Reverse Primer*	*PCR Alleles*
Intron 1	DdeI	5’ GGGACCACTGTGGTATAATGTGG 3’ 5’ CTGGAGGATAGATGTCTCTATCTG 3’	369 bp and 319 bp
Intron 3	XmnI	5’AATCAGAGACTGCTGATTGACTT 3’ 5’ AAACAAGCCAGATAAAGCCTCCA 3’	222 bp and 154+68 bp
Intron 4	TaqI	5’ CTGGAGTATGACTGGCCAATTATCC 3’ 5’ GGTACACAAGGATTCTAAGGTTG 3’	163 bp and 124+39 bp

Genomic DNA was extracted and the markers were processed according to the methods specified by Bowen et al.^11^ The product was run on a 2.5% agarose gel and visualized under UV light.

### DNA sequence analysis

Targeted sequencing was carried out in all the study participants. Corresponding F9 regions were amplified and sequencedon an ABI Prism^®^ 3500 Genetic Analyzer (Applied Biosystems, USA).

### DATA analyses

The data was primarily recorded on Microsoft Excel Spread sheets (Microsoft, USA). Statistical analyses were performed using IBM SPSS^®^ (version 22). The threshold for significance was set at a p-value of 0.05. Allele frequencies and heterozygosity rates for the three RFLPs were determined.

## RESULTS

We investigated a total of 78 individuals, 30 males and 48 females, from 13 families. The participants included 16 known HB patients, 10 obligate carriers, 36 potential carriers and 16 healthy individuals.

Four families were found to be heterozygous for *TaqI* polymorphism; five were heterozygous for *XmnI* polymorphism while three had *DdeI* polymorphism. In four cases the families were heterozygous for both *TaqI* and *XmnI*. *DdeI* heterozygosity was found in isolation in three families. Taken together, the patterns were conclusive in only three (23%) families and were able to define carrier status in only four (11%) of 36 potential carriers. *DdeI* polymorphism established definitive carrier status in two families while the other two polymorphisms (*TaqI* and *XmnI*) could do so in only one family. Allele frequencies and heterozygosity rates for the three polymorphisms in the 13 families are presented in Table-II.

**Table-II:** Allele frequencies and heterozygosity rate of F9 polymorphisms in Pakistani population.

	*Haemophilia B Families*			

*Marker*	*N*	*+*	-	*Het (n)*
Dde1	39	0.11	0.89	0.07 (1)
XmnI	39	0.11	0.89	0.07 (1)
TaqI	39	0.11	0.89	0.15 (2)

Het: heterozygosity rate; N: total number of alleles; n: number of individuals.

**Fig. 1:**
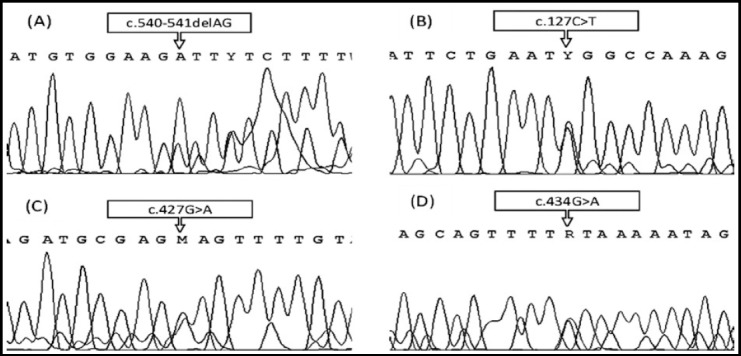
Chromatograms of four randomly selected carriers from different haemophilia B families. **Note:** (A) Mutation c.540-541delAG causing a shift in the sequence frame is expressed in the carrier as an array of dual peaks starting at mutation site. (B) (C) & (D) Heterozygous pattern is marked as dual peaks at mutation sites marked by arrow.

Sanger sequencing was performed in all study participants and a definitive picture was obtained in all the cases. Among 36 potential carriers, 21 were found to have inherited the disease allele and were hence diagnosed as carriers. Fig.1 depicts chromatograms of randomly selected carriers from the study.

BS was calculated in each female participant. The frequent bleeding complaints included menorrhagia, post-partum haemorrhage, prolonged bleeding after tooth extraction and post-surgical bleeding. The mean bleeding score was significantly higher (p value <0.001) in carriers (0.12±0.332) than the normal females (1.48±1.93). It was further elucidated that the mean bleeding score in carriers of 40 years and above age group (3.67±2.2) was significantly raised (p value 0.001) in comparison to that of carriers from age group younger than 40 years (0.96±1.4). None of the study participant, however, had an abnormally raised BS, i.e. > 6 in adult women and >3 in children.

Mean FIX:C level in HB carriers was found to be 79.5 ± 22.2. This was significantly lower (p value <0.001) when compared with a mean FIX:C level of 112.6 ±15.4 found in healthy female relatives. Mean FIX: C level in carriers of <40 years age group (FIX:C 78.9 ±24.5) was statistically similar (p value 0.7) to that of ≥40 years age group carriers (FIX:C 81.8 ±7.5).

**Fig. 2:**
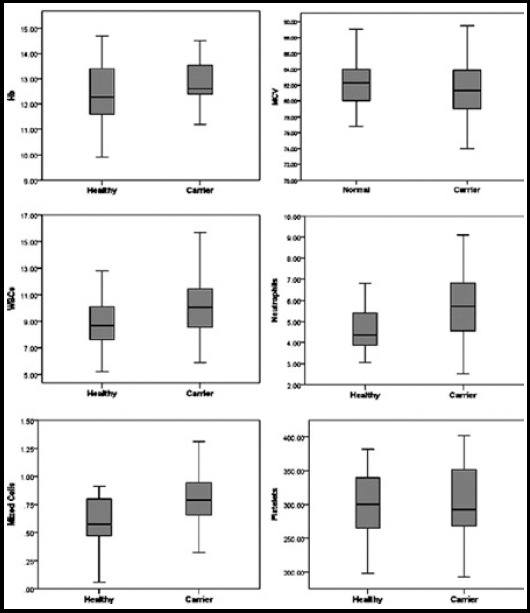
Box plots of selected haematological parameters in healthy and carrier females from HB families. X-axis represents the two groups (healthy and carrier) whereas Y-axis represents the study parameter. Hb, Haemoglobin; MCV, Mean corpuscular volume; WBCs, White blood cells.

The mean white blood cell (WBC) count among carriers (10.33±2.61 x 10^9^/l) was significantly raised (p value 0.025) in comparison to that of healthy female group (8.62 ±2.08 x 10^9^/l). It was further found that the mean neutrophil count of carrier group (5.85 ± 1.96 x 10^9^/l) was higher (p value 0.031) than that of healthy females (4.66 ± 1.37 x 10^9^/l). Mixed cells was also found to vary among the two groups (p value 0.014). The mean mixed cells count was 0.854±0.34 x 10^9^/l in carriers and 0.58 ± 0.36 x 10^9^/l in healthy females (Fig.2). No significant difference between the two groups was found in rest of the CBC parameters.

## DISCUSSION

The study primarily aimed at determining heterozygosity rates for three polymorphisms in HB families from KP and FATA. The secondary objectives included phenotypic assessment of HB carriers. The low frequency of *F9* RFLPs shown in the current study discourages their usage as DNA markers for linkage analysis and carrier identification in the Pakistani HB population. A previous study conducted by Imran et al using 5 different *F9* RFLPs including *DdeI*, found a combined heterozygosity of 70% in Pakistani HB families.^8^ Increased allele frequencies and higher heterozygosity rate has been reported for the *DdeI* in the neighboring Indian population.^6^,^9^ Our findings are, however, consistent with those in Chinese and general Asian populations which report a lower heterozygosity rate.^12^

Direct sequence analysis provides definitive assessment of carrier status in HB.^7^ In the present study the procedure was conclusive in all the study participants. *F9* is a short gene, so sequence analysis and data interpretation can be performed in a short time at a low cost. It is therefore recommended to employ this technique in determining carrier status, where feasible.

BS provides an insight into the bleeding severity in an individual.^13^ In the current study, bleeding scores were found to be significantly raised among the carriers, particularly those above 40 years age. It has been previously established that FIX:C levels increase with age.^14^ In our study, however, the two age groups had a comparable mean FIX:C activity. The difference in BS among the two age groups of HB carriers, therefore, pertains to factors other than FIX:C. We hence suggest a study that could explore these factors in the elderly carriers.

The red blood cell (RBC) indices did not significantly vary among the carrier and normal groups. This is not in concordance with the considerably higher BS in the carrier group. Menorrhagia renders a female susceptible to iron deficiency, which in turn may lead to anemia if not managed well.^15^ We could not identify any other study describing haematological parameters in haemophilia carriers. A further exploration of RBC indices, supplemented with iron status in larger groups of haemophilia carriers is suggested.

In the present study a higher WBC count is found in the carriers as compared to healthy females. There are studies which advocate a uniform WBC count throughout the menstrual cycle in healthy females, yet there are others which report significant fluctuation in the counts.^16^ In this study the neutrophil and mixed cell counts are significantly higher in the carriers. Although obvious diseases were excluded in the questionnaire, exclusion of subclinical infections was not carried out. The exact counts, in various phases of menstrual cycle, ideally together with sex hormones assessments, in larger carrier groups is recommended for a clearer picture.

## CONCLUSION

The study concluded that the three RFLP polymorphisms (*DdeI*, *XmnI* and *TaqI*) have low heterozygosity rates in HB families from KP and FATA and hence have limited diagnostic value. Direct sequence analysis remains a conclusive diagnostic modality. FIX:C activity in carriers is significantly lower than in healthy individuals; the reciprocal is true about BS. The mean WBC count, particularly neutrophil and mixed cell counts, is considerably higher in the HB carriers than the normal females.
